# Molecular characterization and expression analysis of pitaya (*Hylocereus polyrhizus*) *Hp*LRR genes in response to *Neoscytalidium dimidiatum* infection

**DOI:** 10.1186/s12870-020-02368-6

**Published:** 2020-04-15

**Authors:** Min Xu, Cheng-Li Liu, Yu Fu, Zhi-Wen Liao, Pan-Yang Guo, Rui Xiong, Yu Cheng, Shuang-Shuang Wei, Jia-Quan Huang, Hua Tang

**Affiliations:** Hainan Key Laboratory for Sustainable Utilization of Tropical Bioresources, College of Tropical Crops, Hainan University, No.58 Renmin Avenue, Haikou, 570228 Hainan People’s Republic of China

**Keywords:** Pitaya, Canker disease, *Neoscytalidium dimidiatum*, Transcriptomics, Leucine-rich-repeat genes, Expression analysis, qRT-PCR

## Abstract

**Background:**

Canker disease caused by *Neoscytalidium dimidiatum* is a devastating disease resulting in a major loss to the pitaya industry. However, resistance proteins in plants play crucial roles to against pathogen infection. Among resistance proteins, the leucine-rich repeat (LRR) protein is a major family that plays crucial roles in plant growth, development, and biotic and abiotic stress responses, especially in disease defense.

**Results:**

In the present study, a transcriptomics analysis identified a total of 272 LRR genes, 233 of which had coding sequences (CDSs), in the plant pitaya (*Hylocereus polyrhizus*) in response to fungal *Neoscytalidium dimidiatum* infection. These genes were divided into various subgroups based on specific domains and phylogenetic analysis. Molecular characterization, functional annotation of proteins, and an expression analysis of the LRR genes were conducted. Additionally, four LRR genes (CL445.Contig4_All, Unigene28_All, CL28.Contig2_All, and Unigene2712_All, which were selected because they had the four longest CDSs were further assessed using quantitative reverse transcription PCR (qRT-PCR) at different fungal infection stages in different pitaya species (*Hylocereus polyrhizus* and *Hylocereus undatus*), in different pitaya tissues, and after treatment with salicylic acid (SA), methyl jasmonate (MeJA), and abscisic acid (ABA) hormones. The associated protein functions and roles in signaling pathways were identified.

**Conclusions:**

This study provides a comprehensive overview of the *Hp*LRR family genes at transcriptional level in pitaya in response to *N. dimidiatum* infection, it will be helpful to understand the molecular mechanism of pitaya canker disease, and lay a strong foundation for further research.

## Background

Plants suffer from various biotic and abiotic stresses during growth and development, and one of the most important stresses is disease caused by pathogens. Once attacked by a pathogen, plants perceive and recognize the pathogen/microbe-associated molecular patterns (PAMPs/MAMPs) via cell surface receptors and trigger an immune response, which is known as PAMP or MAMP-triggered immunity (PTI/MTI) [1]. In pathogenic microorganism, flagellin (flg22), elongation factor Tu (EF-Tu), peptidoglycan (PGN) and lipopolysaccharide (LPS) from bacterial, with chitin, chitosan from fungal and β-glucans from oomycetes are typical PAMPs [2]. Once attacked by pathogens, plant first to activate defenses by plant pattern recognition receptors (PRRs) which located in cell surface. Surface-localized receptor kinases (RKs), especially the TM-LRR are the most known PRR proteins. One kind of the RKs is surface-localized receptor and the other are receptor-like proteins. These proteins can detect conserved PAMPs by various ligand-binding ectodomains to activate plant PTI/MTI [3]. Compared with PRR-RKs, the PRR-RLPs proteins have the same overall structures but lack an intracellular kinase domain.

In plants, there are two kinds of transmembrane receptor-like kinases (PRR-RLKs) are well-known. One kind of PRR-RKs are receptor-like serine/threonine kinases and the other are receptor histidine kinases. The RLKs are a large family of well-studied kinases with an extracellular domain, a transmembrane domain and an intracellular kinase domain in generally [4]. RLKs are sub-classed to leucine-rich repeat (LRR), lysine motifs receptor kinase (LYK) and *Catharanthus roseus* RLK1-like (CrRLK1L) according to its N-terminal extracellular domain and the leucine-rich repeat receptor like kinases (LRR-RLKs) were the best studied [5]. RLKs are cell surface localized and can recognize distinct ligands of microbial origin. For example, the flg22 and EF-Tu elf18 are detected by flagellin sensitive 2 (FLS2) and elongation factor Tu receptor (EFR) respectively which belong to LRR-RLKs. The chitin elicitor receptor kinases (CERK1) through homodimerization and phosphorylation mediate chitin-induced signaling [6]. The LysM-RK lysine motif receptor kinase5 (LYK5) binds fungal chitin to form a chitin inducible complex with CERK1 to induce plant immunity in Arabidopsis [7].

Not all kinases have extracellular or transmembrane domains, such as receptor-like cytoplasmic protein kinases (RLCKs). But these kinases also have catalytic domain in intracellular localization similar to RLKs [4]. Botrytis-induced kinase1 (BIK1), a plasma membrane-localized RLCK which defined as a serine/threonine kinase interact with FLS2 and brassinosteroid (BR) insensitive 1-associated kinase 1 (BAK1) to initiate plant immune responses to bacterial flagellin [8]. Another RLCK which named BR-signaling kinase 1 (BSK1) played as a substrate of the brassinosteroid receptor BR insensitive 1 (BRI1) to physically associates with the PAMP receptor FLS2 and positively regulates the PTI [9]. These results investigated that PRRs including receptor kinases (RKs), receptor-like proteins (RLPs) and their interacted kinases or proteins play important roles in plant defense of PTI process.

If PAMP-triggered immunity response is considerable, intracellular immunity is activated. Resistance (R) proteins in plants directly or indirectly recognize pathogen effectors and activate effector-triggered intracellular immunity (ETI). In plants, there is a large leucine-rich repeat (LRR) family of R proteins with the consensus amino acid (aa) sequence motif LxxLxLxxN/CxL (x denotes any aa) [10]. Most LRRs are immune receptors and mainly participate in growth, development, the ETI process of plant–pathogen interactions, and stress defense [11]. During ETI process, secreted virulence effectors are perceived by plants intracellular immune receptors. Among those immune receptors, nucleotide binding site leucine repeat receptors (NBS-LRRs) take up a large part [3]. The NBS-LRR type proteins are classified to toll/interleukin receptor (TIR)-NBS-LRR (TNL) and coiled-coil (CC)-NBS-LRR (CNL), and these are the majority resistance (R) proteins in response to pathogen infection in plants [12, 13].

The LRR family genes are mainly referenced to encode proteins with a leucine-rich repeat (LRR) conserved domain which including leucine-rich repeat receptor/receptor-like kinases (LRR-RKs/RLKs) subfamily, leucine-rich repeat receptor/receptor-like proteins (LRR-RPs/LRR-RLPs) subfamily, F-box/LRR-repeat protein (FBXL) subfamily and nucleotide binding site leucine rich repeat (NBS-LRR) subfamily that generally participated in plants immunity. In the aspect of structure, the main features of the LRR genes are their structural domain of 20–30 aa and their transmembrane domain containing 2–42 LRRs [14]. Previous studies showed that the LRR domain provides a versatile structural framework for protein–protein interactions and allows proteins to act as molecular switches in relation to pathogen recognition and defense activation [15, 16].

A typical LRR-RLK contains a N-terminal extracellular receptor domain, a single-pass transmembrane domain and a C-terminal intracellular kinase domain. The N-terminal domain perceives signals, the single-pass domain anchors the protein within the membrane and the C-terminal kinase domain transduces signals downstream via autophosphorylation [17]. In *Arabidopsis thaliana*, the LRR transmembrane receptor kinase FLS2 is a pattern-recognition receptor that determines the specificity of flagellin 22 (flg22) perception [18]. A *Capsicum annuum* (pepper) LRR protein (*Ca*LRR1) was reported to regulate plant cell death and defense by interacting with two pathogenesis-related proteins (*Ca*PR10 and *Ca*PR4b) and hypersensitive induced reaction 1 protein (*Ca*HIR1) [19]. Another *C. annuum* plasma membrane LRR protein (*Ca*LRR51) acts as a positive regulator in response to bacterial *Ralstonia solanacearum* infection [20].

In the LRR-RLK family, the LRR receptor-like serine/threonine-protein kinase (LRR-STK) subfamily is the largest subfamily. The members play crucial roles in pathogen recognition signaling, the subsequent activation of plant defense mechanisms, and developmental control [21]. BRI1 and BAK1 are a pair of RLKs involved in BR signaling, which regulates plant growth, development, and stress responses [22, 23]. BAK1 is also an LRR-STK that acts as a signaling regulator by interacting with BRI1 in vitro and in vivo in *Arabidopsis* [22]. In contrast to LRR-RLKs, LRR-RPs lack a cytoplasmic kinase domain for initiation of downstream signal transduction in plants [24].

The FBXL subfamily is widely found in animals and plants, and there are approximately 40–50 aa in the N-terminus of the conserved F-box domain [25]. It was reported that F-box proteins are the substrate-recognition subunits of Skp1-Cullin-F-box protein (SCF) ubiquitin ligase complexes [26]. Genome-wide analyses showed that F-box genes respond to salt stress, heavy metals, and drought in soybean and the legume *Medicago truncatula* [27, 28]. Auxin-signaling F-box protein 4 (AFB4), which is similar to the auxin receptor known as Toll/interleukin-1 receptor (TIR1), plays a pivotal role in plant growth, development, and innate immunity, as shown by a combination of physiological, molecular, and genetic approaches [29]. An interesting study by Angot *et.al*. (2006) showed that seven type III secretion system (T3SS) effectors that contain both an LRR domain and an F-box domain promote disease in several host plants. The authors stated that this may be because they hijack their host SCF-type E3 ubiquitin ligases to interfere with the host ubiquitin/proteasome pathway to promote disease [30].

In mammals, FBXL2 was reported to interact with the pool of p85 beta subunits to control the phosphatidylinositol-3-kinase (PI3K) signaling cascade by harnessing the proteasomal degradation process [26]. Immunoprecipitation and tandem mass spectrometry (IP-MS) analysis showed that the fork head box M1 (FoxM1) transcription factor interacted with the FBXL2 protein in gastric cancer [31]. Meanwhile, Chen et al. showed that FBXL2 ubiquitinates Aurora B to inhibit tumorigenesis [32]. Furthermore, research by Tosto et al. showed that FBXL7 overexpression was associated with Alzheimer’s disease in a study that compared Alzheimer’s disease-like transgenic mice to wild-type littermates [33]. These results show that FBXL proteins not only participate in growth, development, and disease in plants but also in mammals.

In plants, the NBS-LRR subfamily can directly associate with pathogen-derived effectors as receptors and indirectly sense effector-mediated modification of other host proteins [34]. Functioning as a molecular switch, NBS is a part of the NB-ARC (“nucleotide binding adaptor shared by APAF-1, resistance proteins, and CED-4”) domain [35]. Activated plant NBS-LRRs trigger a range of immune responses, such as the hypersensitive response (HR), which culminates in death of infected cells [36]. There are two major classes of NBS-LRRs in plants: the coiled-coil motif (CC)-NBS-LRR (CNL) subfamily and the TIR-NBS-LRR (TNL) subfamily [37]. The CC-NBS-LRR subfamily have an N-terminal CC domain, while the TIR-NBS-LRR subfamily have a TIR domain [38]. A wheat CC-NBS-LRR (*Ta*RCR1) was reported to positively contribute to the defense response to the fungal pathogen *Rhizoctonia cerealis* by maintaining reactive oxygen species (ROS) homoeostasis [39]. Another CC-NBS-LRR protein *Pm21* in wheat was proved to confer powdery mildew resistance [40]. In the plant *Nicotiana benthamiana,* overexpression of the novel fungal *Plasmoparaviticola*-induced TIR-NBS-LRR gene (*Va*RGA1) enhanced disease resistance and drought and salt tolerance [41]. Soybean resistance was improved to several mosaic virus strains by overexpression of soybean TIR-NBS-LRR type R gene GmKR3 [42]. Similar to other LRR genes, NBS-LRR genes play important roles in plant disease defense signaling.

Pitaya (*Hylocereus polyrhizus* and *Hylocereus undatus*) is an important tropical-subtropical fruit tree found in Central America, East Asia and Southeast Asia. Canker disease caused by the fungi *Neoscytalidium dimidiatum* is one of the most destructive and economically important diseases of in the pitaya industry [43, 44]. At present, due to the lack of reports on the pitaya genome, it is economic and efficient to study biotic and abiotic stress response genes using a high-throughput approach.

The fungal which named *Neoscytalidium dimidiatum* caused canker disease of pitaya has been isolated and identified in our previous work in Hainan Province, China [44]. Transcriptome-wide high-throughput RNA-Sequencing (RNA-Seq) about the expression profiles of resistant genes related to *Neoscytalidium dimidiatum* defense in normal and diseased stem tissues were made ensue [45]. Here, we analyzed and studied the pitaya (*Hylocereus polyrhizus*) LRR receptor-like (*Hp*LRR) family resistant genes in response to *N. dimidiatum* infection based on previous RNA-Seq data detail. Transcriptomics, molecular characterization, and expression results regarding LRR subfamilies in pitaya after *N. dimidiatum* infection have not been previously reported. This study provides an overview of the subfamily classification of LRR genes in pitaya and provides a basis for future functional studies. The evolutionary history and functions of each LRR subfamily in pitaya remains to be understood in future research.

## Results

### Identification and phylogenetic analysis of pitaya *Hp*LRR transcriptional genes

**Fig. 1:**
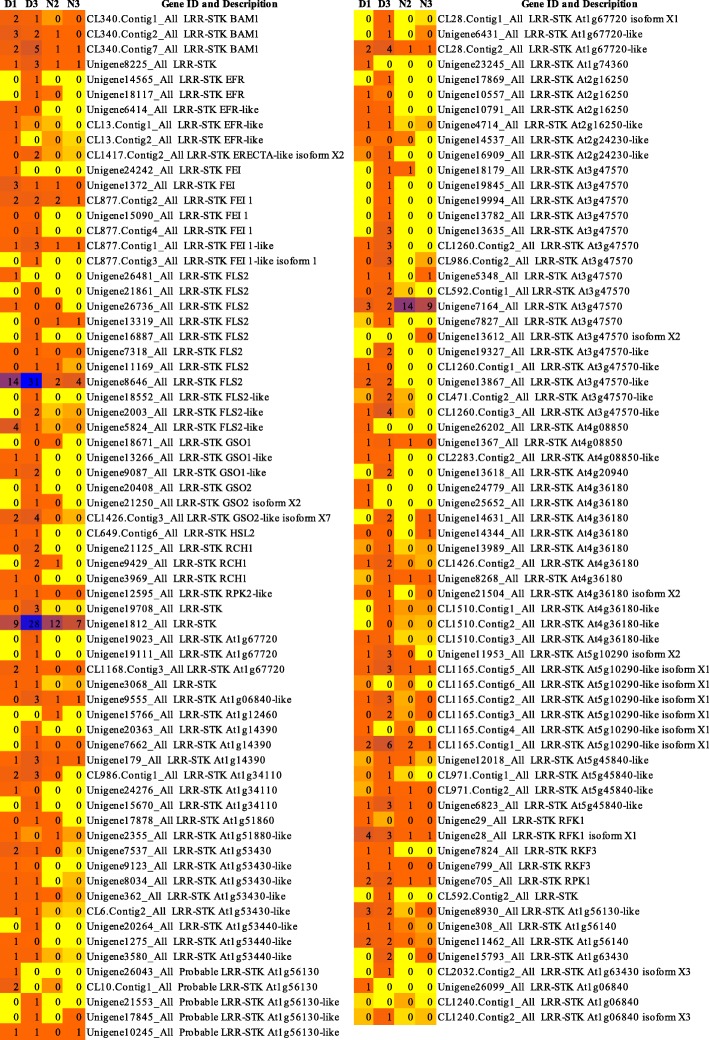
Expression profiles of LRR-STK genes in four samples. N2: normal sample 2; N3: normal sample 3; D1: diseased sample 1; D3: diseased sample 3. LRR-STK: leucine-rich repeat receptor-like serine/threonine protein kinase

**Fig. 2:**
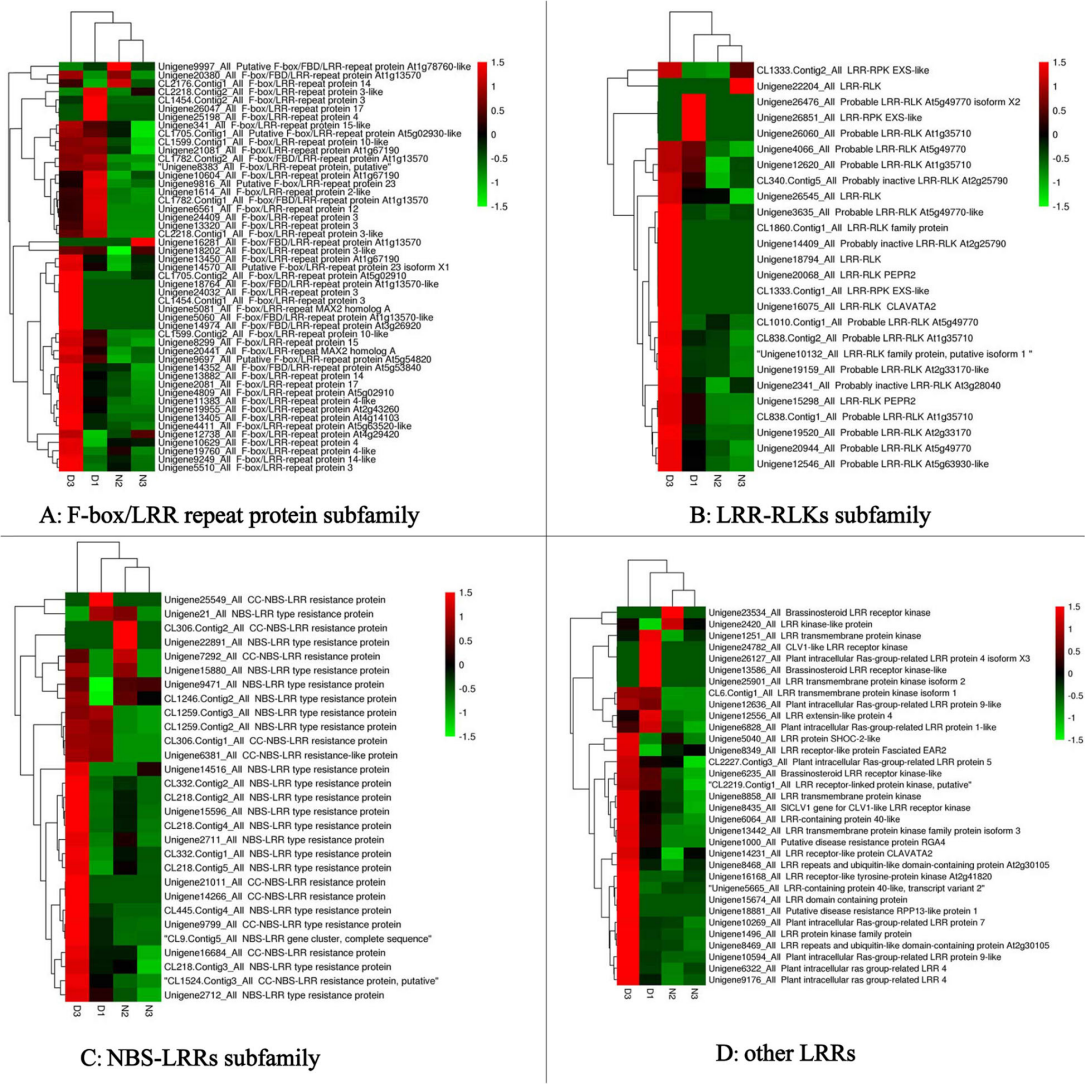
Expression levels of FBXL, LRR-RLK, NBS-LRR, and other LRR subfamilies in four samples. N2: normal sample 2; N3: normal sample 3; D1: diseased sample 1; D3: diseased sample 3. FBXL: F-box/LRR-repeat protein; LRR: leucine-rich repeat; NBS: nucleotide-binding site; RLK: receptor-like kinase

We have obtained the differentially expressed Hiseq data induced by pitaya canker disease through RNA-seq reported in detail in our previous study [45]. In this study, a total of 272 *Hp*LRR transcriptional genes were identified based on previous de novo transcriptomic analysis. The heatmap analysis indicated that most LRR genes were up-regulated in diseased pitaya tissues. Among these genes, 12 (Unigene19327_All, Unigene21125_All, Unigene13635_All, CL1260.Contig2_All, CL2218.Contig1_All, Unigene19955_All, Unigene13405_All, Unigene18881_All, Unigene9087_All, Unigene13867_All, Unigene15298_All, and Unigene12636_All) were significantly up-regulated based on a log2FoldChange (D/N) ≥1.0 (FDR ≤0.001) (Supplemental Material 1). The 272 *Hp*LRR transcriptional genes underwent a BLAST analysis (Supplemental Material 1) involving six protein databases (nr/nt, SwissProt, KEGG, COG, InterPro, and GO). Based on the BLAST analysis, these genes were annotated as belonging to the LRR-STK subfamily (135), FBXL subfamily (49), NBS-LRR subfamily (29), LRR-RLK subfamily (26), plant intracellular Ras group-related LRR (PIRL) subfamily (8), LRR transmembrane protein kinase subfamily (5), brassinosteroid LRR receptor kinase subfamily (3), and other LRR genes (17) (Table 1). Heatmaps of the 272 *Hp*LRR gene expression levels in D1, D3, N2 and N3 samples are presented in Figs. 1 and 2. Although LRR-STK genes belong to the LRR-RLK subfamily, in order to distinguish them from common LRR-RLK genes, separate heatmaps were constructed.

**Table 1:** Classification of LRRs family genes based on six protein databases and phylogenetic analysis.

Based on Six Protein Databases (Total 272)	Classification	Number	Up regulation	Description of Classification
	I	135	Unigene19327_All, Unigene21125_All, Unigene13635_All, Unigene9087_All, Unigene13867_All, CL1260.Contig2_All	LRR-STK subfamily
	II	26	Unigene15298_All	LRR-RLK subfamily
	III	29	---	NBS-LRR subfamily
	IIII	49	CL2218.Contig1_All, Unigene19955_All, Unigene13405_All	FBXL subfamily
	V	8	Unigene12636_All	PIRL subfamily
	VI	5	---	LRR transmembrane protein kinase
	VII	3	---	Brassinosteroid LRR receptor kinase
	VIII	17	Unigene18881_All	Other LRR genes
Based on Phylogenetic Analysis (Total 233)	Classification	Number	aa length	Gene annotation and number
	I	16	31–465	LRR receptor-like/receptor-like STK (9); NBS-LRR (3); FBXL (2); Brassinosteroid LRR receptor kinase (1); LRR transmembrane protein kinase (1)
	II	32	31–927	LRR receptor-like /receptor-like STK (16); FBXL (6); NBS-LRR (6); LRR transmembrane kinase (1); Brassinosteroid LRR receptor kinase (1); PIRL (1); LRR and ubiquitin-like domain-containing protein (1)
	III	24	32–604	LRR receptor-like/receptor-like STK (15); FBXL (3); NBS-LRR (3); PIRL (2); Brassinosteroid LRR receptor kinase-like (1)
	IV	30	31–1138	LRR receptor-like/receptor-like STK (16); NBS-LRR (6); FBXL (5); LRR transmembrane protein kinase (3)
	V	26	34–364	LRR receptor-like/receptor-like STK (24); FBXL (2)
	VI	27	36–501	LRR receptor-like/receptor-like STK (16); FBXL (5); PIRL (4); NBS-LRR (1); Disease resistance protein RGA4 (1)
	VII	24	32–515	LRR receptor-like/receptor-like STK (15); FBXL (7); NBS-LRR (2)
	VIII	54	30–504	LRR receptor-like/receptor-like STK (36); FBXL (11); NBS-LRR (5); PIRL (1); Disease resistance RPP13-like protein (1)

*FBXL* F-box/LRR-repeat protein, *LRR* leucine-rich repeat, *NBS* nucleotide-binding site, *PIRL* plant intracellular Ras group-related LRR, *RLK* receptor-like kinase, *STK* serine/threonine-protein kinase

**Fig. 3:**
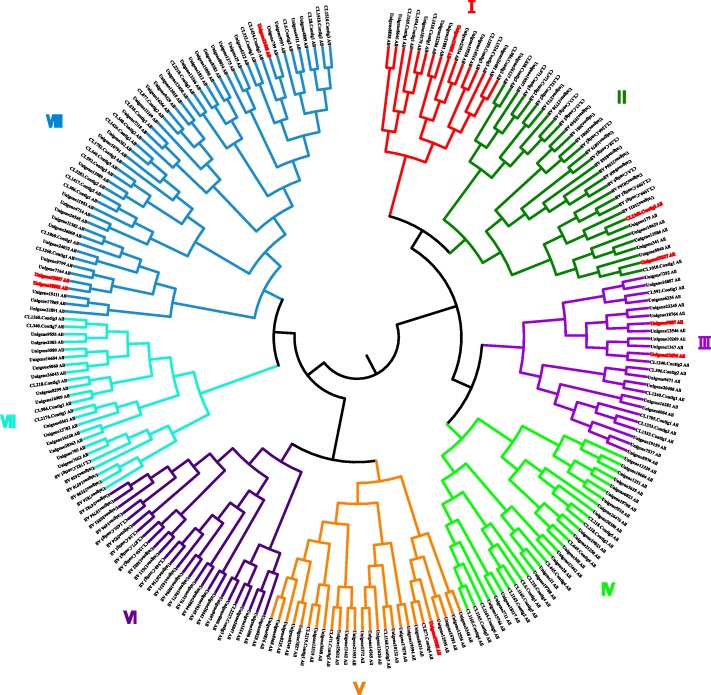
Phylogenetic analysis of 233 *Hp*LRR genes with coding sequences (CDSs) divided to eight subgroups. The 9 red genes are LRR genes that were significantly up-regulated

Among the 272 *Hp*LRR genes, 39 have no CDS, based on base sequence analysis using A plasmid Editor (ApE) software (http://jorgensen.biology.utah.edu/wayned/ape/). In contrast, some *Hp*LRR genes had multiple CDSs. The 33 *Hp*LRR genes with CDSs > 1.0 kb were selected for gene annotation and gene structure analysis, while the four *Hp*LRR genes with the longest CDSs were selected for further expression analysis (at different fungal infection stages, in different pitaya tissues, and after plant hormone treatment). The aa sequence and molecular weight of the 233 *Hp*LRR genes with CDSs varied greatly, from 30 aa/3.382 kDa to 1138 aa/128.777 kDa (Supplementary Material 2). The aa sequences of the 233 *Hp*LRR genes were used for phylogenetic analysis using MEGA 6.0 [46], and the nine significantly up-regulated genes with CDSs are marked in red (Fig. 3). The phylogenetic analysis showed that the 233genes could be divided into eight subfamilies (Fig. 3 and Table 1). This indicated that these genes have some degree of aa-level similarity, suggesting evolutionary relationships. Nevertheless, the base and aa sequence evolutionary results were not consistent (Table 1), due to the degeneracy of codons.

### Gene structure of the 33 *Hp*LRR transcriptional genes with CDSs > 1.0 kb

**Fig. 4:**
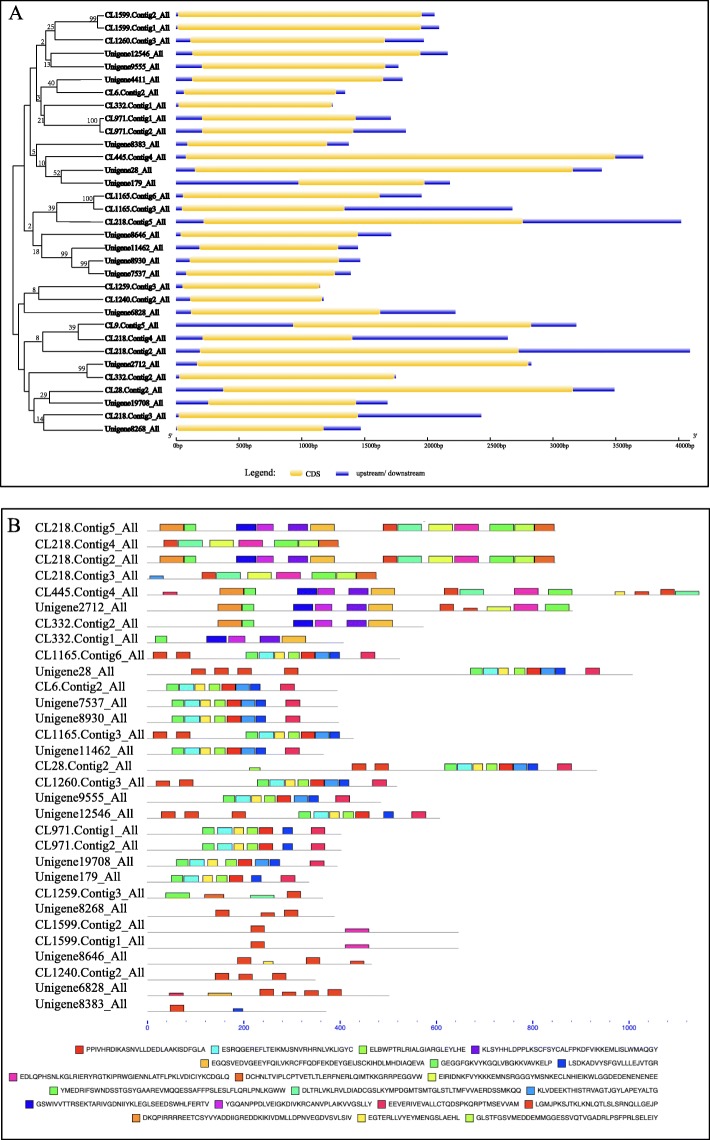
Gene structure and conserved motifs analysis of the 33 pitaya *Hp*LRR transcriptional genes with coding sequences (CDSs) > 1.0 kb. **a** Gene structures: Yellow represents the gene CDS and blue represents the upstream or downstream gene sequences. **b** Conserved motifs of the CDS. The conserved motifs were using the MEME program. Different motifs were highlighted with different color boxes

It was hard to conduct further research on all the LRR genes as there were many of them. Hence, we selected genes with CDSs > 1.0 kb for gene structure analysis and conserved motifs analysis (Fig. 4) for further study. The detailed information of these 33 genes with CDSs > 1.0 kb was showed in Table 2. All 33 *Hp*LRR transcriptional genes have upstream and downstream sequences. CL1599.Contig2_All and CL1599.Contig1_All; CL971.Contig1_All and CL971.Contig2_All; CL1165.Contig6_All and CL1165.Contig3_All; Unigene11462_All, Unigene8930_All, and Unigene7537_All; and Unigene2712_All and CL332.Contig2_All have 99–100% similarity, indicating that each set of two or three genes have close evolutionary relationships.

**Table 2:** Information of the 33 pitaya *Hp*LRR transcriptional genes with CDSs > 1.0 kb

Unigene	Length	CDS	N -FPKM	D -FPKM	log2FoldChange(D/N)	Annotation (nr, SwissProt, KEGG, COG, InterPro, GO)
CL445.Contig4_All	3716	3417	0.49	1.77	1.85	NBS-LRR type resistance protein
Unigene28_All	3386	3006	0.88	3.82	2.13	Probable LRR receptor-like STK RFK1 isoform X1
CL28.Contig2_All	3487	2784	0.65	2.89	2.15	Probable LRR receptor-like STK At1g67720-like
Unigene2712_All	2825	2636	1.39	3.10	1.15	NBS-LRR type resistance protein
CL218.Contig5_All	4019	2529	0.62	0.82	0.42	NBS-LRR type resistance protein
CL218.Contig2_All	4089	2529	0.78	1.41	0.85	NBS-LRR type resistance protein
CL1599.Contig2_All	2057	1938	0.81	1.82	1.17	FBXL 10-like
CL1599.Contig1_All	2093	1938	0.26	0.64	1.29	FBXL 10-like
Unigene12546_All	2161	1815	0.47	2.1	2.16	Probable LRR receptor-like protein kinase At5g63930-like
CL332.Contig2_All	1749	1714	1.05	2.34	1.16	NBS-LRR type resistance protein
CL1165.Contig6_All	1953	1566	0.12	0.12	0.00	Probable LRR receptor-like STK At5g10290-like isoform X1
CL1260.Contig3_All	1971	1548	0.09	2.45	4.76	Probable LRR receptor-like STK At3g47570-like
Unigene4411_All	1802	1515	0.23	1.15	2.35	FBXL At5g63520-like
Unigene6828_All	2223	1506	0.79	1.7	1.11	PIRL 1-like
Unigene9555_All	1769	1449	0.87	1.56	0.85	Probable LRR receptor-like STK At1g06840-like
CL218.Contig3_All	2429	1428	0.48	0.64	0.43	NBS-LRR type resistance protein
Unigene8646_All	1712	1398	3.05	22.85	2.91	LRR receptor-like STK FLS2
CL9.Contig5_All	3183	1389	1.18	4.88	2.05	NBS-LRR gene cluster, complete sequence
CL1165.Contig3_All	2675	1281	0.33	1.15	1.80	Probable LRR receptor-like STK At5g10290-like isoform X1
CL332.Contig1_All	1245	1219	0.96	1.33	0.48	NBS-LRR type resistance protein
CL971.Contig1_All	1708	1203	0.05	0.71	3.97	Probable LRR receptor-like STK At5g45840-like
CL971.Contig2_All	1828	1203	0.48	0.50	0.04	Probable LRR receptor-like STK At5g45840-like
CL218.Contig4_All	2640	1194	0.82	1.70	1.05	NBS-LRR type resistance protein
Unigene8930_All	1465	1188	0.26	2.41	3.24	Probable LRR receptor-like STK At1g56130-like
Unigene7537_All	1389	1182	0.22	1.84	3.09	Probable LRR receptor-like STK At1g53430
CL6.Contig2_All	1344	1179	0.11	0.86	2.96	Probable LRR receptor-like STK At1g53430-like
Unigene19708_All	1682	1179	0.05	1.78	5.15	Putative LRR receptor-like STK
Unigene8268_All	1469	1167	0.74	0.76	0.04	Probable LRR receptor-like STK At4g36180
Unigene8383_All	1373	1113	0.25	1.03	2.07	Putative FBXL
Unigene11462_All	1447	1095	0.26	1.89	2.86	Probable LRR receptor-like STK At1g56140
CL1259.Contig3_All	1145	1091	1.09	4.54	2.06	NBS-LRR type resistance protein
CL1240.Contig2_All	1174	1049	0.10	0.68	2.75	Probable LRR receptor-like STK At1g06840 isoform X3
Unigene179_All	2179	1005	1.16	1.79	0.63	Probable LRR receptor-like STK At1g14390

*N -FPKM* Fragments per kilobase of transcript per million mapped reads (FPKM) of normal samples (N); *D -FPKM* Fragments per kilobase of. transcript per million mapped reads (FPKM) of disease samples (D). *FBXL* F-box/LRR-repeat protein; LRR: leucine-rich repeat; NBS: nucleotide-binding site; *PIRL* Plant intracellular Ras group-related LRR; *RLK* Receptor-like kinase; *STK* Serine/threonine-protein kinase

### Verification of 12 differentially expressed LRR genes (DEGs) by qRT-PCR

Based on a PossionDis analysis, 12 genes were significantly up-regulated (|log2FoldChange (D/N)| ≥ 1.0, FDR ≤0.001) among the total set of 272 LRR genes. Among these 12 genes, seven (Unigene15298_All, Unigene21125_All, Unigene13635_All, CL1260.Contig2_All, Unigene13867_All, Unigene19327_All, and Unigene9087_All) were annotated as belonging to the LRR-STK subfamily, three (CL2218.Contig1_All, Unigene19955_All and Unigene13405_All) as belonging to the FBXL subfamily, one (Unigene18881_All) as a disease resistance gene, and one (Unigene12636_All) as belonging to the PIRL subfamily.

**Fig. 5:**
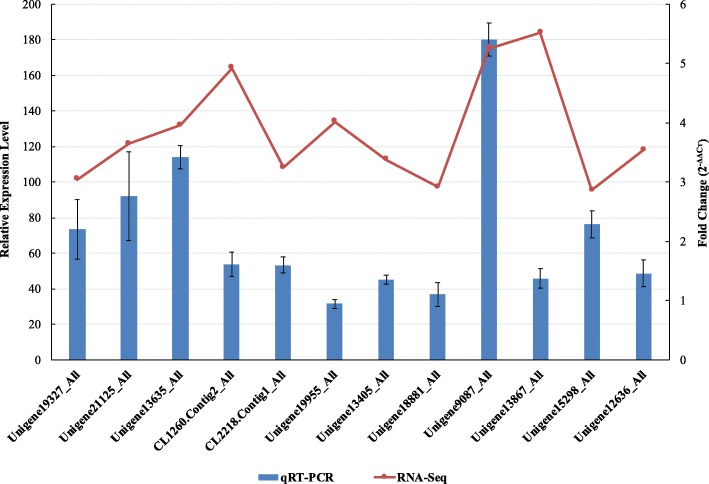
Verification of 12 differentially expressed LRR genes (DEGs) by qRT-PCR assays. Gene expression levels were measured by qRT-PCR and compared with RNA-Seq results. The histogram represents the fold changes of genes (diseased [D]/normal [N]) according to qRT-PCR, and the line chart represents gene expression according to RNA-Seq. All genes selected for qRT-PCR analysis were analyzed using three biological replicates. Error bars represent ±SD (2^-ΔΔCт^) based on three experiments

To verify the RNA-Seq results, qRT-PCR assays were performed. The qRT-PCR expression level trends of 11 of the 12 genes (not Unigene19955_All) were consistent with the RNA-Seq results (Fig. 5). In addition, three of the 12 (Unigene15298_All, Unigene21125_All, and CL2218.Contig1_All) have no CDS, and the number of aa of the remaining nine genes ranged from 35 to 290 (Supplementary Material 2).

### Expression profiles of four *Hp*LRR genes under different stages of *N. dimidiatum* infection in different pitaya species

**Fig. 6:**
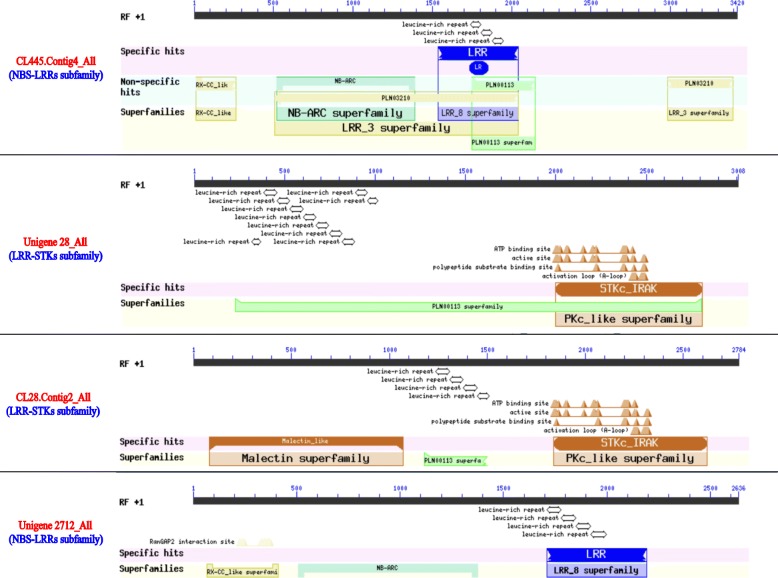
Conserved domains of the four LRR genes used in further research. Annotations: CL445.contig4_All and Unigene2712_All: NBS-LRR; Unigene28_All and CL28.contig2_All: LRR-STK. LRR: leucine-rich repeat; NBS: nucleotide-binding site; STK: serine/threonine-protein kinase

Four *Hp*LRR genes (CL445.Contig4_All, Unigene28_All, CL28.Contig2_All, and Unigene2712_All) were selected for the subsequent expression analysis as they had the four longest CDSs (Supplementary Material 2). The conserved domain analysis indicated that CL445.Contig4_All and Unigene2712_All were NBS-LRR type resistance proteins, while Unigene28_All and CL28.Contig2_All were probable LRR-STKs (Fig. 6). The four genes have 3–9 LRR motifs (Fig. 6). In addition, CL445.Contig4_All and Unigene2712_All belong to the RX-CC_like family (“Coiled-coil domain of the potato virus X resistance protein and similar proteins”) and the NB-ARC family. Unigene28_All and CL28.Contig2_All have specific hits regarding the STKc_IRAK family (“Catalytic domain of the serine/threonine kinases, interleukin-1 receptor associated kinases, and related STKs”) and a common LRR-RLK (the PLN00113 superfamily). CL28.Contig2_All has a specific malectin-like domain; malectin is a novel endoplasmic reticulum carbohydrate-binding protein and a candidate player in the early steps of protein N-glycosylation [47].

**Fig. 7:**
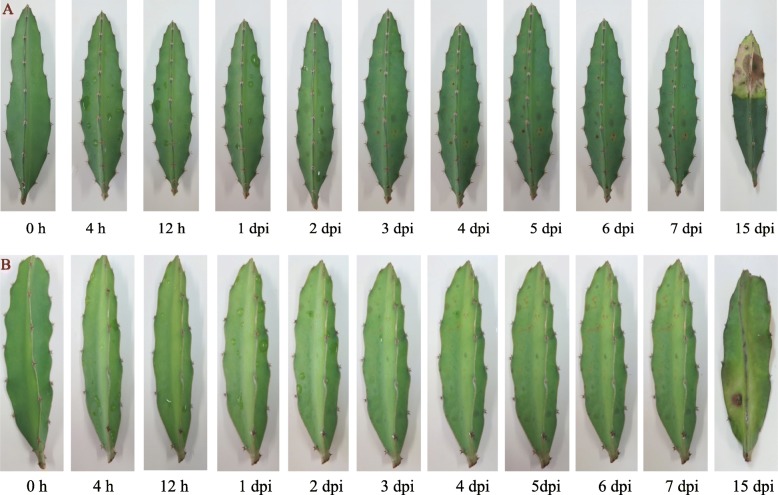
Symptoms of different stages of *N. dimidiatum* infection of pitaya. Dpi: days post infection. **a**: Red-fleshed pitaya (*Hylocereus polyrhizus*) infected by *N. dimidiatum*; **b**: white-fleshed pitaya (*Hylocereus undatus*) infected by *N. dimidiatum*

**Fig. 8:**
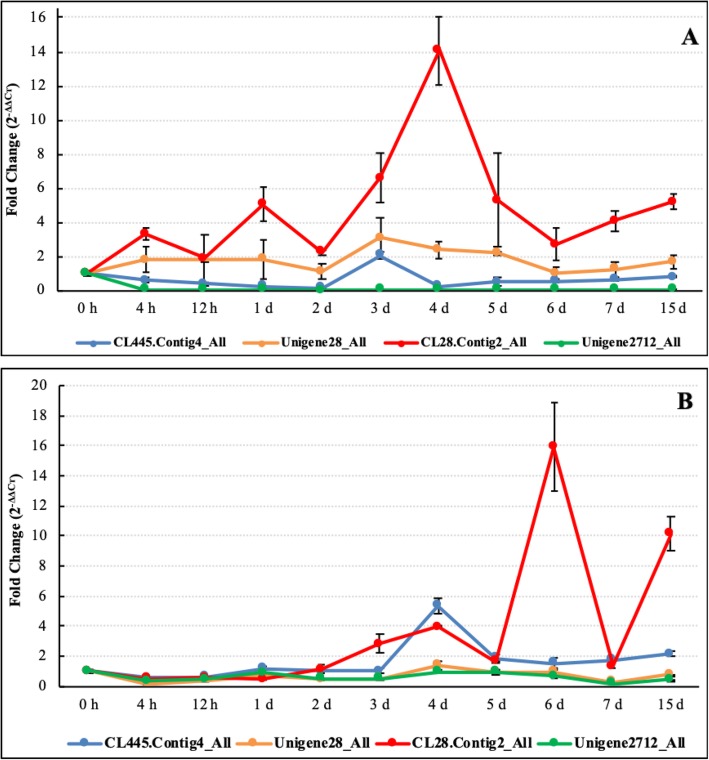
Expression profiles of four *Hp*LRR transcriptional genes at different stages and different pitaya species of *N. dimidiatum* infection. (**a**): *Hp*LRR genesexpression profiles in red-fleshed pitaya (*Hylocereus polyrhizus*) infected by *N. dimidiatum*; (**b**): *Hp*LRR genes expression profiles in white-fleshed pitaya(*Hylocereus undatus*) infected by *N. dimidiatum*. The X-axis represents different stages of *N. dimidiatum* infection and the Y-axis represents the foldchange (2^-ΔΔCт^) according to qRT-PCR. Annotations: CL445.Contig4_All and Unigene2712_All: NBS-LRR; Unigene28_All and CL28.Contig2_All: LRRSTK. LRR: leucine-rich repeat; NBS: nucleotide-binding site; STK: serine/threonine-protein kinase

The pitaya stems presented obvious symptoms after 3 days of *N. dimidiatum* infection and rotted by day 15 (Fig. 7). However, compared with red-fleshed pitaya (Fig. 7a), the white-fleshed pitaya (Fig. 7b) possessed strong resistance. Due to the protective hard wax coat on the pitaya stems, the expression levels of the four LRR genes peaked after 3~4 days of infection in red-fleshed pitaya while 4~6 days in white-fleshed pitaya. The expression results (Fig. 8a) suggested that, in red fleshed pitaya (*Hylocereus polyrhizus*), compared with control level (0 h), CL445.Contig4_All expression first decreased and then suddenly increased on day 3, then decreased, and finally exhibited an increasing trend but remained lower than the control level. Unigene28_All and CL28.Contig2_All expression levels were increased after *N. dimidiatum* infection, but Unigene28_All reached a peak on day 3 while CL28.Contig2_All reached a peak on day 4. In contrast to the other three genes, Unigene2712_All expression always decreased compared with the control level. These results indicated that the four genes participated in the fungal infection response of pitaya, especially CL28.Contig2_All, which was significantly up-regulated on day 4. In contrast to the other three genes, Unigene2712_All may act as a negative regulator in plant–pathogen interactions. In the white-fleshed pitaya (*Hylocereus undatus*), the genes expression has the similarity trend. However, the infection time of the genes reached a peak in white-fleshed pitaya was later 1 day compared with red-fleshed pitaya (Fig. 8b). The four *Hp*LRR genes expression files results were consistent with the infection symptoms of the two different pitaya species (Fig. 7).

### Tissue-specific expression profiles of the four *Hp*LRR genes

**Fig. 9:**
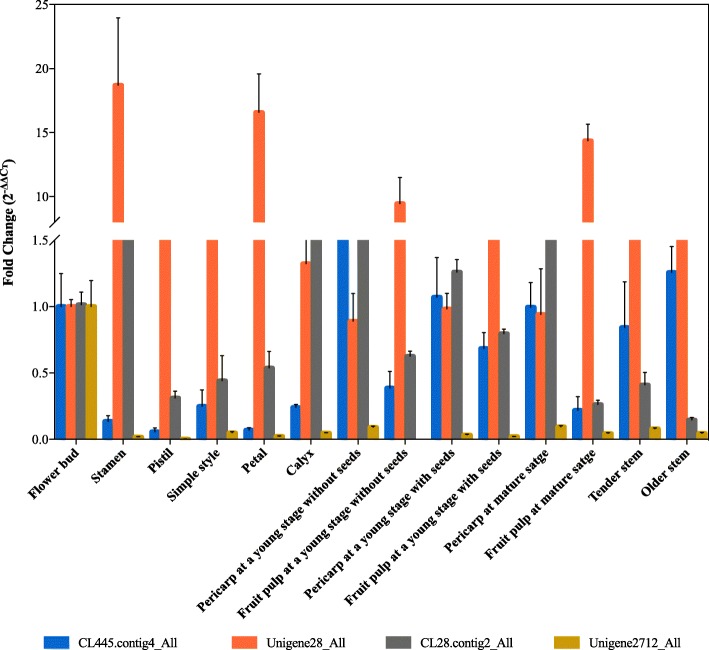
Expression profiles of four *Hp*LRR genes in different pitaya tissues. The X-axis represents different pitaya tissues and the Y-axis represents the fold change (2^-ΔΔCт^) according to qRT-PCR. Annotations: CL445.contig4_All and Unigene2712_All: NBS-LRR; Unigene28_All and CL28.contig2_All: LRR-STK. LRR: leucine-rich repeat; NBS: nucleotide-binding site; STK: serine/threonine-protein kinase

Tissue-specific genes (also known as luxury genes) are genes whose products have specific functions in specific cell types. To investigate whether the four *Hp*LRR genes we selected have specific functions in specific cells, tissue-specific expression profiles were obtained by qRT-PCR for 14 pitaya tissues (Fig. 9). CL445.Contig4_All was mainly expressed in the pericarp of a young green fruit; Unigene28_All was mainly expressed in the stamen, petal, and fruit pulp of both a young green fruit and a red fruit; CL28.Contig2_All was significantly expressed in the pericarp of both a young green fruit and a red fruit, and Unigene2712_All was mainly expressed in the flower bud. These results showed that Unigene28_All was significantly up-regulated and may play pivotal roles in pitaya flower and fruit growth and development.

### Expression profiles of the four *Hp*LRR genes in response to SA, ABA, and MeJA treatments

**Fig. 10:**
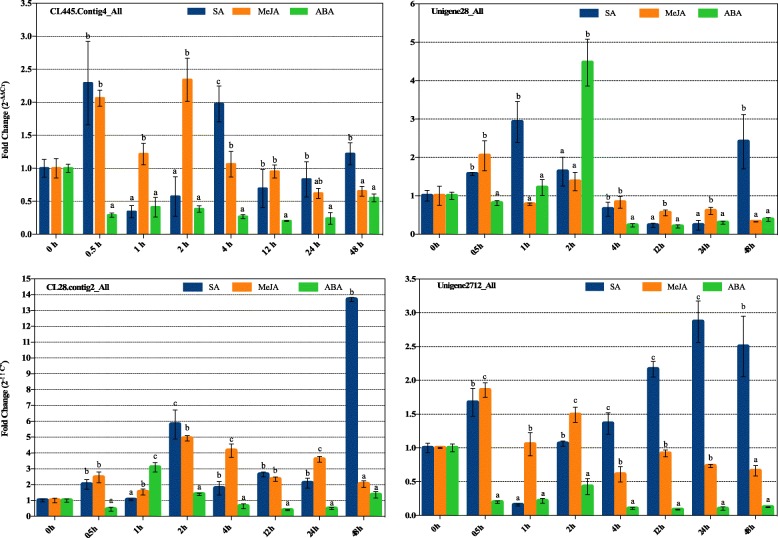
Expression profiles of four *Hp*LRR genes in response to SA, MeJA and ABA treatment. Annotations: CL445.contig4_All and Unigene2712_All: NBS-LRR; Unigene28_All and CL28.contig2_All: LRR-STK. LRR: leucine-rich repeat; NBS: nucleotide-binding site; STK: serine/threonine-protein kinase. Different letters (a, b, c) indicate significant difference of the expression of the target gene based on three technological replications [*P* < 0.05, Single factor ANOVA test (F test), *n* = 3]

In plants, hormones play important roles in response to a wide range of biotic and abiotic stress signaling networks. SA, ABA, jasmonates (JAs), and ethylene have crucial well-known roles in plant disease and pest resistance [48]. To better understand the four *Hp*LRR genes’ responses to hormonal regulation of the plant–pathology interaction pathways, the expression patterns of these genes in response to SA, ABA, and MeJA treatments were assessed by qRT-PCR (Fig. 10). All four genes responded to the three hormones to some degree. Unigene28_All expression was significantly changed after 2 h of ABA treatment. CL28.Contig2_All was prominently expressed at 48 h of SA treatment. Unigene2712_All was significantly down-regulated by ABA, but significantly up-regulated by SA, reaching a peak at 24 h. The results showed that Unigene28_All and CL28.Contig2_All may play pivotal roles in the hormone-mediated disease resistance response.

## Discussion

In the present study, we identified 272 LRR genes in pitaya in the de novo transcriptome assembly analysis, and 233 of them have CDSs. Most of these genes belonged to the LRR-RLK, NBS-LRR, or FBXL subfamilies and, within the LRR-RLK subfamily, the LRR-STK subfamily had the largest number of genes. The results of annotation and conserved domain analysis showed that LRR genes have the typical LxxLxLxxNxL domain and most of which were participated in plant disease. Despite having distinct evolutionary origins, most animals and plants need cell-surface or intracellular immune receptors with an LRR domain to detect pathogens and trigger defense responses [49]. In mammals, LRR kinases play key roles in disease, especially Parkinson’s and Crohn’s diseases [50, 51]. In plants, most of these LRRs are resistance proteins and can directly associate with pathogen-derived effectors or indirectly sense effector-mediated modification of other host proteins [52]. Diverse classes of cell-surface immune LRR receptors share important features for the initiation of resistance. The LRR genes identified in this study may play crucial roles in the pitaya immune response. The phylogenetic analysis showed that the base and aa sequence results were not consistent (Table 1) due to the degeneracy of codons. Additionally, genes with similar base sequences do not necessarily have similar functions.

Besides the three large LRR subfamilies, we also found eight PIRL genes, five LRR transmembrane protein kinase genes, three brassinosteroid related LRR receptor kinase genes, and 17 other LRR genes, such as genes for CLAVATA (CLV)-like LRR receptor kinases, the LRR protein SHOC-2-like, and the LRR receptor-like protein FASCIATED EAR2 (Fig. 2). PIRLs are a plant-specific class of LRR proteins that take part in developmental cell signaling and gene regulation, for example, in *A. thaliana*, PIRL1 and PIRL9 have redundant roles that are essential at a key transition point early in pollen development [53].

Most LRR-RLKs are transmembrane kinase and phosphorylate serine/threonine residues (also known as LRR-STK), and most LRR-RLKs is recognition of an extracellular ligand, which leads to activation of the intracellular kinase domain and subsequent transduction of downstream signaling pathways [54]. The five LRR transmembrane protein kinases genes that we identified may belong to the LRR-RLKs subfamily.

It has been reported that the LRR-RLK gene BAK1/SERK3 is involved in brassinosteroid signaling and is a major modulator of PAMP-triggered immunity in *A. thaliana* and *N. benthamiana* [55]. The three brassinosteroid LRR receptor kinase genes in this study may play critical roles in the brassinosteroid signaling pathway in response to *N. dimidiatum* infection of pitaya.

It has been reported that CLV1 is an RLK composed of an LRR-containing extracellular domain (with putative receptor function) and a cytoplasmic STK domain (linked via a transmembrane domain); it is thought to play an important role in meristem maintenance and flower development [54, 56]. Compared with CLV1, CLV2 is structurally similar but with a very short predicted cytoplasmic tail [57]. CLV3 is a stem cell-specific protein that activates the *Arabidopsis* CLAVATA stem cell signaling pathway [58]. These reports indicate that CLV proteins mainly participate in the growth and development of plants.

Four genes (CL445.Contig4_All, Unigene28_All, CL28.Contig2_All, and Unigene2712_All) were selected (because they had the four longest CDSs) for a further expression analysis. Among them, CL445.Contig4_All and Unigene2712_All were NBS-LRR resistance proteins, while Unigene28_All and CL28.Contig2_All were probable LRR receptor-like STK genes (Fig. 6). CL28.Contig2_All was significantly up-regulated on day 4 after *N. dimidiatum* infection while Unigene2712_All was significantly down-regulated in all infection stages. However, the time of the gene expression reached a peak in white-fleshed pitaya was later 1 day than that of red-fleshed pitaya (Fig. 8b). These two genes may play positive or negative roles in plant disease responses. Unigene28_All was significantly expressed on day 3 after *N. dimidiatum* infection and, based on the tissue-specific expression profiles, also significantly expressed in the stamen, petal, and fruit pulp of both a young green fruit and a red fruit. These results showed that Unigene28_All not only participates in pitaya flower and fruit growth and development but also in the disease resistance response. In addition, the expression levels of the four LRR genes peaked 3~4 days post infection in red-fleshed pitaya while 4~6 days in white-fleshed pitaya, suggested that it probably displayed different resistance among different pitaya species.

Plant hormones, which include SA, JAs, ABA, brassinosteroid, ethylene, and auxin, act as signals to trigger and mediate plant immune responses [59]. In plants, SA is a secondary metabolite with a central role as a signaling molecule in the activation of plant resistance to pathogens [60, 61]. Research has shown that SA accumulation was increased in infected leaves and uninfected leaves that mediated systemic acquired resistance (SAR) [62]. SA accumulation can parallel the increase in pathogenesis-related protein gene expression [61]. For example, BAK1 overexpression caused SA accumulation and deregulation of cell death control genes [63].

JAs, including jasmonic acid and the MeJA derivatives, have proved effective at improving plant stress tolerance [64]. It was reported that JAs are key signaling compounds in hypersensitive response cell death and accumulate in response to bacterial flg22 in the grape *Vitis rupestris* [65].

Similar to SA and JAs, ABA (a weak acid that contains 15 carbon atoms) is an important stress hormone and is involved in physiological processes [66]. ABA participates in embryogenesis, seed germination, floral transition, and so on [67, 68].

**Fig. 11:**
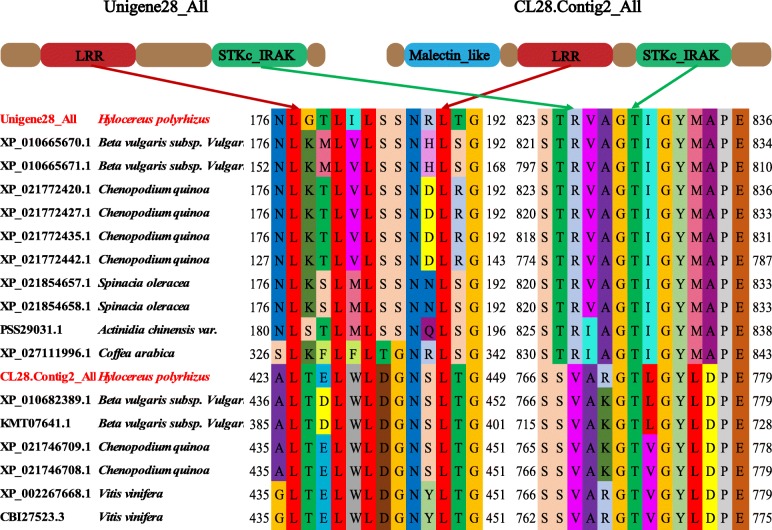
Conservation of functional motifs in non-angiosperm species of two genes (Unigene28_All and CL28.Contig2_All). Red arrows represent the typical conservation of LRR, while green arrows represent the typical conservation of the STKc_IRAK conserved sites. The representation of the domains is approximate and not to scale

However, plant growth, development, and stress responses need multiple phytohormones involved in complex signaling network crosstalk [69]. In this study, pitaya stems were treated with SA, MeJA, and ABA. Although all four genes responded to the three hormones, CL28.Contig2_All expression reached a peak at 48 h after SA treatment and Unigene28_All was clearly regulated by ABA at 2 h. The results of hormones treatments showed that the expression of LRR family genes was regulated by plant hormones. The LRR family resistant genes and hormones related genes probably form a cross network to regulated plant disease together. Taking all the expression results together, we selected these two genes for further functional research. The subsequent analysis of conserved functional motifs in Unigene28_All and CL28.Contog2_All indicated that these two genes both have the typical LxxLxLxxNxL domain and the STRVGTIGYMAPE or SSVAxGTL/VGYLDPE conserved sites of STKc-IRAK (x denotes any aa) (Fig. 11). These domains or conserved sites may play important roles in protein function and signaling. However, in some cases, an LRR protein requires another helper or partner protein for functionality, so a complete functional study (including screening for interacting proteins) regarding Unigene28_All and CL28.Contog2_All is being carried out and the results will be published in the future.

Canker disease caused by *N. dimidiatum* is one of the most serious diseases of pitaya in the main growing regions [44]. The *N. dimidiatum* is a fungal which has a strong specificity infection of its host pitaya. Currently, because there is a very hard technological bottleneck in genetic transformation system of pitaya, it is very difficult to investigate the gene function in pitaya by transgenic methods just like in model plants. The fungal also can’t infect the model plants like *Arabidopsis thaliana*, tobacco and tomato by our exploratory testing. So, it is a very hard work to identify the genes function with really deep experiments. At present, there are no reports on transcriptomics, molecular characterization, and expression analyses of LRR genes in pitaya induced by *N. dimidiatum* infection. From the expression profiles, we can identify the *Hp*LRR genes in response to pitaya canker disease preliminary. These results provide important research information for a relatively poor studied pathosystem at this moment, provide a framework for further functional investigation of the pitaya LRR subfamilies, and contribute to a better understanding of the complexity of LRR genes in higher plants.

## Conclusions

Canker disease caused by the fungus *N. dimidiatum* is one of the most important diseases because of its rapid spread and strong specificity infection in its host pitaya. The occurrence of canker disease not only poses a significant threat in yields but also affects the quality of pitaya. Currently, it is an important job to excavate the resistant genes and explain the functions of them. This study mainly analyzed the *Hp*LRR family resistant genes structures by bioinformatics analysis and expression profiles in different pitaya tissues, different infected stages of red-fleshed pitaya (*Hylocereus polyrhizus*) and white-fleshed pitaya (*Hylocereus undatus*), and plant hormone treatments of *Hp*LRR family resistant genes. Results of this study provided a comprehensive overview of the *Hp*LRR family genes at transcriptional level in pitaya in response to *N. dimidiatum* infection and provided a basis for further in-depth functional studies.

## Methods

### Plant materials and growth conditions

The normal (N1, N2, and N3) and diseased (D1, D2, and D3) tissues of pitaya (*Hylocereus polyrhizus*) stems used for RNA-seq were collected from Ledong County, Hainan Province, China that had been grown for about 5 years under natural conditions on a plantation. The normal (N) group were healthy plants while the disease (D) group were diseased plants infected by *N. dimidiatum* fungal. The pitaya stems and fruits which infected by *N. dimidiatum* fungal appear small yellow spot initially, then spread quickly and almost all of the stems will go to rot finally. De novo transcriptome assembly and RNA-Seq were conducted using an Illumina HiSeq System by Shenzhen BGI Tech Company (Shenzhen, China). Principal component analysis (PCA) and correlations analysis were used to evaluate the repeatability of samples. After analysis, the N1 and D2 samples were abandoned due to poor repeatability. Therefore, the bioinformatics analysis and follow-up experiments were carried out based on four samples (N2, N3, D1, and D3). The accession number of RNA-sequencing data and profiles was GSE119976 in Gene Expression Omnibus database of NCBI. In addition, the pitaya stem tissues for *N. dimidiatum* fungal infection, hormone treatment and tissues specific expression were collected in a plantation of Hainan University not same with those used for RNA-seq. Five pitaya plants were used in each condition and displayed similar symptoms. All five pitaya plants were merged together to use for qRT-PCR experiments.

### Sequences assembled and RNA-Seq bioinformatics analysis

Clean data was used for bioinformatics analysis which obtained by filtering from sequenced raw data [45]. Trinity paired-end assembly method was used to identify pitaya *Hp*LRR family genes [70]. TIGR Gene Indices clustering tools was used to obtain unigenes by clustering the assembled clean reads and eliminating redundancy [71]. These unigenes were blasted to public proteins databases to annotate and analyze. Which including. The Expectation Maximization (RSEM) software (Version: V1.2.12; Parameter: default; http://deweylab.biostat.wisc.edu/ RSEM) was used to obtain expression level expressed in FPKM. The calculation method of unigenes’ FPKM was described as Li B [72]. The up- or down-regulated unigenes with an adjusted *P*-value ≤0.05 and fold change |(log2FC) ≥ 1| were defined as differentially expressed genes (DEGs) using NOIseq and PossionDis methods.

### Identification and phylogenetic analysis of pitaya *Hp*LRR transcriptional genes

The assembled unigenes were blasted to nr, GO, KEGG, COG, InterPro and SwissProt databases and the unigenes annotated to LRR family genes were selected for the next analysis. The expression levels of all *Hp*LRR genes were analyzed by heatmap analysis using the free online data analysis platform OmicShare tools (http://www.omicshare.com/tools/, Figs. 1 and 2). Thereafter, all the sequences of 233 LRR genes with coding sequences (CDSs) were used to construct a phylogenetic tree based on 1000 bootstrap replicates and the neighbor-joining (NJ) method using MEGA 6.0 software [46].

### Gene structure and phylogenetic analysis of 33 *Hp*LRR transcriptional genes with CDSs> 1.0 kb

There were 33 *Hp*LRR transcriptional genes with CDSs> 1.0 kb. Gene structure analysis of these genes was performed using the free online platform Gene Structure Display Server (GSDS v2.0, http://gsds.cbi.pku.edu.cn) by uploading the genes’ CDSs and assembly sequences. Conserved motifs analysis was carried on MEME program based on the amino acid sequences of the CDS. The phylogenetic analysis was carried out based on 1000 bootstrap replicates and the neighbor-joining (NJ) method using MEGA 6.0 software [46].

### Verification of 12 significantly up-regulated differentially expressed LRR genes (DEGs) by quantitative reverse transcription (qRT)-PCR

In order to verify the reliability and consistency of the up or down regulated genes, all of the 12 differentially expressed LRR genes based on a PossionDis analysis were selected to perform qRT-PCR assay. The total RNA extraction of N2, N3, D1 and D3 pitaya samples were used improved cetyltrimethylammonium bromide (CTAB) method [73]. First strand cDNA synthesis of RNA was conducted according to manufacturer’s protocols (No.6210A, Takara, Japan) after treated with DNase I (Thermo Fisher Scientific, USA). Then, the N2 and N3 cDNA template were balanced mixed together to obtain total N sample, same with D1 and D3 to obtain total D sample. qRT-PCR assay was performed using a 20-μL reaction system according to the manufacturer’s protocols of ChamQ™ Universal SYBR® qPCR Master Mix kit (Q711–02/03, Vazyme Biotech Co., Ltd., Beijing, China). Twelve significantly up-regulated *Hp*LRR family genes were selected for qRT-PCR assays using a 7500 Applied Biosystems qRT-PCR System (Life Tech, 81 Wyman Street, Waltham, MA, 02454, USA). It was proved that the pitaya ubiquitin gene (*UBQ*) was an great internal reference for data normalization [74]. The 12 *Hp*LRR family genes and *UBQ* primers were designed using Primer Premier 6 software (www.premierbiosoft.com). The qRT-PCR conditions were performed as previous study [45]. The Delta-Delta cycle threshold (2^-ΔΔCT^) method was used to output data analysis based on three technical replicates.

### Expression profiles of four *Hp*LRR transcriptional genes under different stages of *N. dimidiatum* infection in different pitaya species

Among the total set of LRR genes, four *Hp*LRR transcriptional genes (CL445.Contig4_All, Unigene 28_All, CL28.Contig2_All, and Unigene 2712_All) were selected for further expression research as they had the four longest CDSs. Red-fleshed pitaya (*Hylocereus polyrhizus*) “Jinduyihao” and white-fleshed pitaya (*Hylocereus undatus*) “Vietnam Bairou” tender stems were used for *N. dimidiatum* infection. The 5 healthy individual pitaya plants were used as control which collected from a plantation in Hainan University. Another 5 healthy individual pitaya plants were used for infected. The infected experiments were performed as following: *N. dimidiatum* hypha were collected after growing them in potato dextrose agar medium for 7–10 days. Before inoculation, the stems were sterilized twice using 75% ethyl alcohol. The hyphae were diluted, with 20–40 conidia in suspension, using 400X microscope magnification (Olympus Corporation, Tokyo, Japan). Inoculation was conducted by spraying the conidial suspension onto the pitaya stems. After spraying, stems were wrapped in absorbent wool with distilled water to retain moisture. The plants were then placed in an illuminated incubator at 28 °C under a 16/8 h light/dark cycle for 15 days. The pitaya stem tissues were collected for total RNA extraction after 0 h, 4 h, 12 h, 1 day, 2 days, 3 days, 4 days, 5 days, 6 days, 7 days, and 15 days of *N. dimidiatum* infection. The total RNA was reversed transcribed into cDNA and the cDNA was used for qRT-PCR (as in the previous steps). The qRT-PCR primers for the four LRR genes were designed using Primer Premier 6.0 software (www.premierbiosoft.com). The primer details for the four genes are presented in Supplenmentary Information 1, 2, 3, 4.

### Expression profiles of the four *Hp*LRR genes in different tissues of pitaya

The following 14 healthy pitaya tissues were studied: (1) flower bud, (2) stamen, (3) pistil, (4) simple style, (5) petal, (6) calyx, (7–12) pericarp and fruit pulp of a young green, mature green, and red fruit, (13) tender stems, and (14) older stems were collected in pitaya plantation, Hainan province. The total RNA of the 14 tissues was extracted, reversed transcribed into cDNA, and used for qRT-PCR to obtain the expression profiles of the four selected genes.

### Response of the four *Hp*LRR genes to salicylic acid (SA), methyl jasmonate (MeJA), and abscisic acid (ABA) treatments

Healthy pitaya stems were collected from the plantation and sterilized twice using 75% ethyl alcohol before being treated with 5 mM SA solution (pH 5.7), 1 mM MeJA solution, and 100 uM ABA solution. The three solutions were all added with 0.01% Silwet-77 (Code: DE0025, Beijing BioDee Biotechnology Co. Ltd., China) to reduce the water’s surface tension. The stem tissues were collected after 0 h, 0.5 h, 1 h, 2 h, 4 h, 12 h, 24 h, and 48 h of hormone treatment. RNA extraction and qRT-PCR assays were performed as in the previous steps.

## Supplementary information

**Additional file 1 Supplenmentary Information 1:** Information of all of the 272 genes.

**Additional file 2 Supplenmentary Information 2:** Details information about the sequence characteristics of 233 LRR related-genes with the longest CDS.

**Additional file 3 Supplenmentary Information 3:** Primers used for the genes in the manuscript.

**Additional file 4 Supplenmentary Information 4:** Sequences were identified as LRR family genes on each database.

## Data Availability

The accession number of RNA-sequencing data and profiles was GSE119976 in Gene Expression Omnibus (GEO) database via NCBI website (https://www.ncbi.nlm.nih.gov/geo/query/acc.cgi?acc=GSE119976). The datasets generated and analyzed during the current study are available from the GEO website or corresponding author on reasonable request.

## Abbreviations

LRRs: Leucine-rich repeat genes
LRR-RLKs: Leucine-rich repeats receptor-like kinases
NBS-LRRs: Nucleotide binding site leucine-rich repeats
LRR-STKs: Leucine-rich repeats receptor-like serine/threonine-protein kinases
BRI1: Brassinosteroid-insensitive 1
BAK1: BRI1-associated receptor kinase 1
NB-ARC: Nucleotide binding adaptor shared by Apaf-1 resistance proteins, CED-4 domain
CC-NBS-LRR: Coiled-coil motif nucleotide-binding site leucine-rich repeat
SA: Salicylic acid
JA: Jasmonates
MeJA: Methyl jasmonate
ABA: Abscisic acid
SAR: Systemic acquired resistance
PR: Pathogenesis related protein
