# Supplementary material for: Molecular characterization and expression analysis of pitaya (Hylocereus polyrhizus) HpLRR genes in response to Neoscytalidium dimidiatum infection
Source: BMC Plant Biol. 2020 Apr 15;20:160. doi: 10.1186/s12870-020-02368-6 (PMC7161156; doi:10.1186/s12870-020-02368-6)
Supplement: Supplementary file 3 — Additional file 3 Supplenmentary Information 3: Primers used for the genes in the manuscript. [file 12870_2020_2368_MOESM3_ESM.docx]

| **Gene ID** | **Primers** | **Product (bp)** |
| --- | --- | --- |
| Unigene9087_All | F: 5’-CTGATGCGATAGGAAATG-3'  R: 5’-ATCCAAGTTACCGATGCT-3' | 185 |
| Unigene13867_All | F：5’-TCAGCCTCGGGAGTAATAGC-3'  R：5’-GCTTAGGCTGGATGGGATT-3' | 125 |
| Unigene15298_All | F：5’- TAGTGTAAGATTGGGAAGAC -3'  R：5’- ATTAGCAGATGTTATGGGT -3' | 118 |
| Unigene12636_All | F：5’-TGATTTGTCTGGGAGGAAGC-3'  R：5’-CGACTGGAGGGAGTTGGA-3' | 166 |
| Unigene19327_All | F：5’-TATCGGTTACGCTCCTCC-3'  R：5’-TCATCTGTGGGCCTCTTT-3' | 117 |
| Unigene21125_All | F：5’-GAGCAGTGGGAATATGAA-3'  R：5’-AAGTTGCCTCGCTTTACT-3' | 105 |
| Unigene13635_All | F：5’-TCTTTGCCTTGGTTACAT-3'  R：5’-AGTTCCAGTAGGAGGTGTT-3' | 128 |
| CL1260.Contig2_All | F：5’-GGCTGTGGATGCTCTAAG-3'  R：5’-AATAACATGATTGAAGTGGC-3' | 139 |
| CL2218.Contig1_All | F：5’-GGATGCCCTAAATTGAAGC-3'  R：5’-TGTAATCGGCAAATAGGAAAG-3' | 126 |
| Unigene19955_All | F：5’-AATCCATTGCCATTGTCG-3'  R：5’-TCTCCCATTCATCCTCCC-3' | 82 |
| Unigene13405_All | F：5’-TCAACTCGTCGTCGTGGGA-3'  R：5’-GAGGTCCTTATTGTTCTTGCTTCT-3' | 120 |
| Unigene18881_All | F：5’-TTGTTGTGGGCAAGGATA-3'  R：5’-CAGCATCGCTTGGATTAG-3' | 114 |
| *UBQ* | F：5′-TGAATCATCCGACACCA-3′  R：5′-TCCTCTTCTTAGCACCACC-3′ | 192 |

**1. Primers for qRT-PCR validation of 12 up-regulation genes**

| **Gene ID** | **Primers** | **Product (bp)** |
| --- | --- | --- |
| CL445.Contig4_All | F: 5’- GAGCGATTACCGTTGTTT -3'  R: 5’- GCACCACCCACTACCTTC -3' | 155 |
| Unigene 28_All | F：5’- TGTAAGAGGCAGAAGTAGAAG -3'  R：5’- GGATTGAAGTTGTCGGTAG -3' | 102 |
| CL28.Contig2_All | F：5’- CAAGGTCATTGATCCGTAA -3'  R：5’- AGCAACTTCAGCCACTCT -3' | 101 |
| Unigene 2712_All | F：5’- CGTGTTGAGATTTGTTTCCCTG -3'  R：5’- GGCATCCTGCTACCCATG -3' | 143 |

**2. Primers for qRT-PCR validation of the four genes**
